# Prehospital stroke diagnostics based on neurological examination and transcranial ultrasound

**DOI:** 10.1186/2036-7902-6-3

**Published:** 2014-02-27

**Authors:** Moriz Herzberg, Sandra Boy, Thilo Hölscher, Michael Ertl, Markus Zimmermann, Karl-Peter Ittner, Josef Pemmerl, Hendrik Pels, Ulrich Bogdahn, Felix Schlachetzki

**Affiliations:** 1 Department of Neurology, University of Regensburg, Community District Hospital, Universitätsstr.84, Regensburg 93053, Germany; 2 Department of Radiology and Neuroscience, University of California San Diego, San Diego, CA, USA; 3 Department of Emergency Medicine, University Hospital Regensburg, Regensburg, Germany; 4 Department of Anesthesiology, University Hospital Regensburg, Regensburg, Germany; 5 Malteser Rettungsdienst, Regensburg, Germany; 6 Department of Neurology, Krankenhaus der Barmherzigen Brüder Regensburg, Regensburg, Germany

**Keywords:** Acute stroke, Emergency medicine, Prehospital diagnostics, Transcranial neurosonography, Mobile health unit

## Abstract

**Background:**

Transcranial color-coded sonography (TCCS) has proved to be a fast and reliable tool for the detection of middle cerebral artery (MCA) occlusions in a hospital setting. In this feasibility study on prehospital sonography, our aim was to investigate the accuracy of TCCS for neurovascular emergency diagnostics when performed in a prehospital setting using mobile ultrasound equipment as part of a neurological examination.

**Methods:**

Following a ‘911 stroke code’ call, stroke neurologists experienced in TCCS rendezvoused with the paramedic team. In patients with suspected stroke, TCCS examination including ultrasound contrast agents was performed. Results were compared with neurovascular imaging (CTA, MRA) and the final discharge diagnosis from standard patient-centered stroke care.

**Results:**

We enrolled ‘232 stroke code’ patients with follow-up data available in 102 patients with complete TCCS examination. A diagnosis of ischemic stroke was made in 73 cases; 29 patients were identified as ‘stroke mimics’. MCA occlusion was diagnosed in ten patients, while internal carotid artery (ICA) occlusion/high-grade stenosis leading to reversal of anterior cerebral artery flow was diagnosed in four patients. The initial working diagnosis ‘any stroke’ showed a sensitivity of 94% and a specificity of 48%. ‘Major MCA or ICA stroke’ diagnosed by mobile ultrasound showed an overall sensitivity of 78% and specificity of 98%.

**Conclusions:**

The study demonstrates the feasibility and high diagnostic accuracy of emergency transcranial ultrasound assessment combined with neurological examinations for major ischemic stroke. Future combination with telemedical support, point-of-care analysis of blood serum markers, and probability algorithms of prehospital stroke diagnosis including ultrasound may help to speed up stroke treatment.

## Background

Ischemic stroke is a time-critical vascular disease that affects neural function and is the leading cause of permanent disability in people in industrialized nations [1,2]. Although the ECASS 3 trial widened the time window for intravenous (IV) thrombolysis to 4.5 h [3] and this window may be extended in selected patients undergoing interventional thrombectomy [4], the majority of patients do not benefit since fewer than 25% of patients arrive within 2 h of symptom onset [5] and only 36% arrive within 3 h (Bavarian Society for Quality control 2012 report) [6]. Significant prehospital delays are the main reason why patients do not receive effective treatment [2,7,8]. Recent analyses from previous studies demonstrate a total median prehospital delay varying between 35 and 71 min [9,10]. Ideally, this time period may be devoted for diagnostics, early allocation to an appropriate hospital, and initiation of stroke-specific therapies [11-13].

Transcranial color-coded sonography (TCCS) is a feasible, fast, and non-invasive bedside method for the evaluation of cerebral arteries in acute stroke, and it is a routine tool in most stroke units. Particularly when contrast agents are applied, TCCS is valid compared with computed tomography (CT) angiography [14] and magnetic resonance angiography (MRA) [15] for the diagnosis of arterial occlusions in patients with acute ischemic stroke, especially in middle cerebral artery (MCA) obstructions [16]. According to the ‘Neurosonology in Acute Ischemic Stroke study’, TCCS is an independent predictor for stroke patient's outcome [17]. Assessment of vascular pathology and hemodynamics in patients with acute stroke is thought to enable early judgment of functional outcome and thrombolytic efficacy and could identify patients who might benefit from interventional treatment [18-20]. In our study, we focused TCCS examination on the detection of middle cerebral artery occlusion in its proximal segment (M1-MCA occlusions) - the most common site for cerebral artery occlusions - since we hope to shorten time from symptom onset to beginning of therapy with a very early diagnostic approach.

### Goal of this investigation

In this ‘Regensburg stroke mobile project’, we hypothesized that a neurologist equipped with a portable ultrasound device is able to achieve a similar diagnostic accuracy ‘in the field’ as compared with in-hospital advanced neuroimaging (CTA, MRA).

## Methods

### Study design

We describe a single-site prospective study in which we compare the results of preclinical neurological examinations supported by TCCS in the field with the results of standard stroke imaging studies (CTA, MRA) and with final discharge diagnoses from the treating stroke unit. In the hospital, standard stroke care was applied without a dedicated imaging or treatment algorithm. In this regard, prehospital TCCS was performed to confirm or deny the presence of major intracranial artery occlusions and not to detect intracranial hemorrhage. Despite the non-invasive nature of the study, we obtained written informed consent from the patient or the next available relative. The study was approved by the local ethics committee (Ethic committee Nr. 09/135) and was performed in accordance with guidelines set out in the Declaration of Helsinki.

### Setting

**Figure 1:**
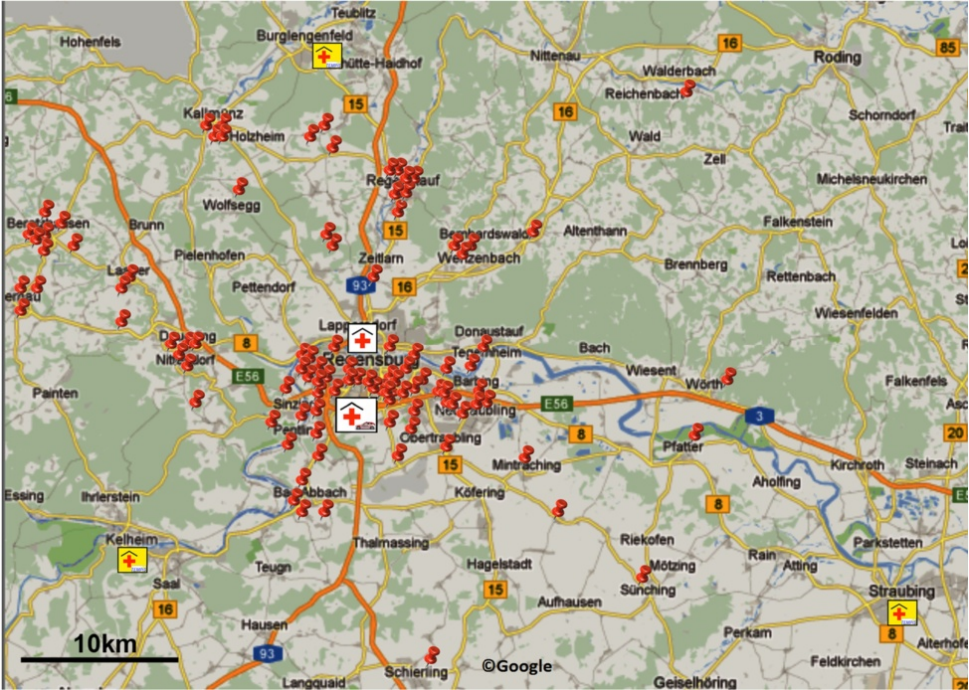
**Map showing Regensburg and the surrounding area.** Pins indicate sites of emergency calls. The Regensburg stroke mobile was housed at the stroke unit of the Department of Neurology, Bezirksklinikum Regensburg. The second stroke unit in Regensburg is located at the Department of Neurology, Krankenhaus Barmherzige Brüder Regensburg. Three telemedical stroke units within the TEMPiS network are located near Regensburg. Bar indicates 10 km (Google©).

The diagnostic portion of the study was performed between May 2010 and January 2011 in the city and rural district of Regensburg. This region supports a population of approximately 150,000 people in east Bavaria, Germany; the operational area that we covered extended up to 35 km in radius (Figure 1).

### Selection of participants

Patient enrollment took place during regular work hours (8 a.m. to 4:30 p.m.), Monday through Friday. Patients were enrolled consecutively and unselected by the dispatch center. The dispatch center did not follow dedicated inclusion criteria but decided following its internal routine algorithms. After the dispatch center received a ‘112’ stroke call (the German equivalent for a ‘911’ stroke call in the USA), the ambulance team (emergency physician and paramedic) and the stroke team (a stroke- and TCCS-experienced neurologist and a paramedic driver in a BMW series 1, dedicated ‘stroke mobile’) were both alerted and sent to the site of the incident. After first aid had been provided to the patient and vital parameters had been stabilized, a brief neurological examination was performed. No TCCS was performed if no neurological symptoms suggestive for acute stroke or transient ischemic attack (TIA) were present. In such cases or if the patient did not show any neurological symptoms, patients either stayed at home or were transferred to the nearest emergency medical department. These cases were not included in the study follow-up. All patients who presented symptoms indicating probable or definite acute stroke were included in this analysis.

### Interventions

In all patients with symptoms of an acute stroke, neurological examination was immediately followed by a TCCS assessment. Neurological examination was based on a simplified and structured assessment including paresis in face, arm, and leg; speech disorders; consciousness; and gaze palsy. Symptoms indicating probable or definite acute stroke were defined as one positive symptom with acute onset. Additionally acute stroke was proposed if the neurologist had the suspicion of stroke due to symptoms like dizziness, hemianopia, and related symptoms.

The highest priority in all cases was to avoid any delay before hospital admittance. Ultrasound examination took place either at the site of the initial treatment (for example, at the patient's couch, on the floor, or at bedside) or during ambulance transport. All neurological patients were transferred to a specialized stroke unit. All patients underwent emergency diagnostic examinations consisting of non-contrast brain CT and, if necessary, CTA and MRA. The primary vascular diagnostic method was chosen based on the patient's level of consciousness, comorbidities (for example, a cardiac pacemaker was a contraindication for magnetic resonance imaging (MRI)), and severity of symptoms. The final diagnosis was made by the responsible stroke team neurologist based on all available clinical information and the contents of the patient's medical record. Patients in whom imaging studies provided evidence of cerebral infarction were given a final diagnosis of ischemic stroke, even if their neurological deficits were transient. A diagnosis of TIA was given to patients in whom deficits lasted less than 24 h, and there was no imaging evidence of infarction. To allow a comparison between standard imaging methods and TCCS, patients who received IV thrombolysis were only included if they had undergone at least one vascular imaging study before IV thrombolysis.

### Ultrasound equipment and data acquisition

We used two portable color duplex ultrasound machines equipped with a phased array transducer capable of transcranial imaging: SonoSite Micromaxx® with a P17 transducer (SonoSite Inc., Bothell, WA, USA) and Philips CX50® with a P2-5 transducer (Philips Ultrasound, Bothell, WA, USA). The standard setting with a transmission frequency of 2.0 MHz for brightness, color, and Doppler mode was used on both machines. Images were stored as bitmap (Micromaxx®) and DICOM (CX50®) files on the hard drive and converted later to jpg files for data transfer and off-line analysis.

An ultrasound contrast agent (UCA; SonoVue®, Bracco Imaging SpA, Milan, Italy) was administered intravenously via a peripheral vein primarily in cases in which the quality of the transcranial bone window was deemed inferior and an urgent diagnosis needed. Intravenous injections of 0.5 to 2 ml UCA were administered, depending on the quality of the temporal bone window, as previously described [21]. After identification of the best temporal bone window, the protocol required color-mode visualization and confirmatory flow measurements in the proximal M-1 segment of both MCAs using spectral Doppler ultrasonography. Angle correction was not performed. The examiner could decide whether to extend the protocol to measurements in the anterior and posterior cerebral arteries (ACAs and PCAs, respectively). Proximal MCA occlusions were diagnosed when the ipsilateral ACA and/or the contralateral ACA and MCA could be visualized and imaging confirmed the existence of a sufficient temporal bone window with or without UCA. Distal MCA or MCA branch occlusions were defined according to criteria published by Zanette and coauthors [22]. The TCCS examination time was defined as the time between the first and last image, as documented in the imaging files. Pathological disorders of the internal carotid artery (ICA) were suspected in either the absence of ipsilateral ACA and MCA flow or a reversal of flow in the ACA that was suggestive of >80% stenosis or total occlusion of the ICA [23]. All TCCS examinations were reviewed by an experienced sonographer who is certified by the German Society of Ultrasound in Medicine (FS, DEGUM Stage III).

### Outcome - primary end point

Primary end point of this study is accuracy of TCCS compared to the ‘gold standard’ neurovascular imaging (CTA/MRA).

### Outcome - secondary end point

Secondary end points include accuracy of initial working diagnosis compared to discharge diagnosis. Safety aspects are side effects of contrast agent.

### Primary data analysis

A simplified data collection sheet was used, which notes the timing of emergency call, arrival at the patient's side, and patient handover to hospital staff; timing of ultrasound examination and whether visualization of both MCAs had been achieved; final diagnosis after the patient was discharged from the hospital; and documentation of stroke treatment used. Data derived from neurovascular imaging studies, such as CTA, MRA, or in-house neurosonography, were collected and correlated to the results of the prehospital TCCS study. The distance from base hospital to the patient was calculated by the navigational system, and values are given as median values with standard deviations.

### Sensitivity analyses

Based on the clinical and TCCS data leading to a prehospital diagnosis and the final discharge diagnosis, we calculated the sensitivity, specificity, and positive and negative predictive values as well as the respective 95% confidence intervals (CIs) of the procedure in determining stroke vs. mimic. Based on the TCCS data, we calculated the same statistics for determining occlusion of the MCA. All data were entered into an Excel worksheet and calculated using MedCalc (version 11.6.1; http://www.medcalc.org).

## Results and discussion

### Results

#### *Characteristics of study subjects*

Table 1 lists baseline demographic characteristics. We received 232 emergency calls and rendezvoused with the first aid team at the patient's site. We excluded 119 patients because their initial clinical examinations did not show stroke or neurological symptoms but instead suggested other disorders. These patients were not examined using TCCS. Another 11 patients were excluded after the emergency doctor and stroke neurologist excluded acute stroke plus TCCS indicated normal intracranial arterial flow. These patients were transferred to general emergency departments, and some were in hospitals without a stroke unit or even stayed at home. There was no follow-up on these patients. The time used to perform the ultrasound examination was 5 min and 36 s. (mean, SD ± 2 min and 12 s).

**Table 1:** Baseline characteristic of the study sample and examiner, location, and time to ultrasound

**Characteristic**	**Value**
All patients, *n*	232
Patients included, *n*	102
Sex (female/male)	54/48
Mean age (SD)	76.8 (13.41)
Ultrasound examination time (mean, SD)	5 min, 36 s (2 min,12 s)
Alarm-to-handover duration (mean, SD)	65 min (25 min)
Contrast enhanced TCCS, *n*	41
Distance to hospital, km	10 (2–41)
Clinic admission, *n* = 102	
Stroke unit	98
Internal Medicine	3
Telemedicine Stroke unit	1
Examining physician	
Investigator 1	57
Investigator 2	42
Investigator 3	3
Site of ultrasound investigation	
Patient's home	51 (50%)
During transport in ambulance car	43 (42%)
Private office practice	4 (4%)
Public space	2 (2%)
Senior citizen home	2 (2%)

TCCS, transcranial color-coded sonography; SD, standard deviation.

#### *Stroke diagnosis - overall sensitivity and specificity*

**Figure 2:**
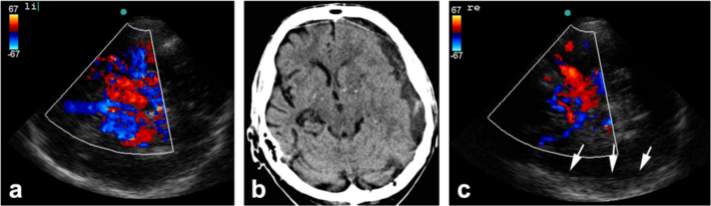
**Left-sided subdural hematoma in an 80-year-old patient. (a)** TCCS performed 30 min after onset of sensory aphasia shows a patent left MCA and the complete circle of Willis. **(b)** Cranial CT scan demonstrates a subdural hematoma (SDH), and **(c)** when viewed in retrospect, TCCS from the right side reveals the SDH in the contralateral hemisphere in B-mode (arrow). The patient's medical history included hypertension, diabetes, and a fall 2 days earlier.

**Figure 3:**
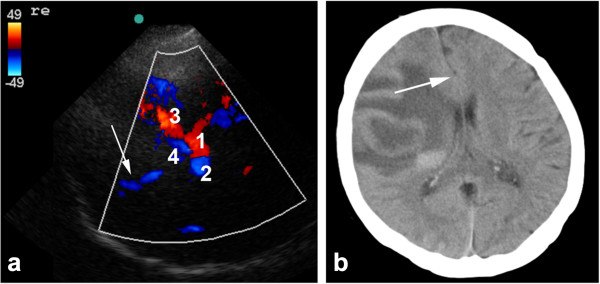
**Images obtained in a 54-year-old patient with progressive left-sided paralysis due to a brain tumor. (a)** TCCS revealed patent arteries, but the midline has shifted as seen in a non-optimal temporal bone window during CT. (1) ipsilateral and (2) contralateral posterior cerebral artery, (3) ipsilateral middle cerebral artery, and (4) ipsi- and contralateral anterior cerebral arteries **(b)** with shift to the contralateral side (arrow).

Of the 102 patients included in the study, 73 (72%) received a confirmed diagnosis of stroke by their treating hospital neurologists and 29 (28%) were correctly classified as stroke mimics. In the field, 4 patients were given the misdiagnosis of a non-stroke event (4%), whereas 15 patients (15%) received the misdiagnosis of stroke when their symptoms merely mimicked those of a stroke. In summary, the initial working diagnosis prior to patient admission to the hospital showed a sensitivity of 95% (95% CI 86 to 98) and a specificity of 48% (29 to 67) in the hospital workup (Tables 2, 3, and 4). Two examples of stroke mimics with interesting neurosonographic findings (normal flow but indications for subdural hematoma or midline shift) were found in a patient with a subdural hematoma (Figure 2) and a brain tumor (Figure 3). In 68% of the patients, stroke-like symptoms were caused by ischemic stroke/TIA with suspected etiology of large artery atherosclerosis in 50% followed by cardioembolism and small vessel disease (Table 5). Only 5% of symptoms were caused by any intracranial hemorrhage. During the study period, 9 of 50 patients (18%) received IV thrombolysis and 1 patient underwent mechanical thrombectomy.

**Table 2:** Initial working diagnostic

**Stroke (*n*** **= 102)**	**Stroke mimics**
Proved right (*n* = 69)	*n* = 3 exsiccosis
	*n* = 2 hypoglycemia
	*n* = 2 syncope
	*n* = 1 pneumonia
	*n* = 1 migraine
	*n* = 1 slipping
	*n* = 1 persisting atrial fibrillation
	*n* = 1 functional brachiofacial hemiparesis
	*n* = 1 hypertensive rise
	*n* = 1 epileptic seizure
Proved wrong (*n* = 4)	*n* = 5 epileptic seizure
	*n* = 4 tumor
	*n* = 2 subdural hematoma
	*n* = 1 exsiccosis
	*n* = 1 MI + brain concussion
	*n* = 1 metabolic encephalopathy
	*n* = 1 peripheral nerve compression (C7)

**Table 3:** Preclinical working and discharge diagnostics

		**Discharge diagnostic**		
		**Stroke**	**Stroke mimic**	**Total**
Preclinical working diagnostic	Stroke	69	15	84
	Stroke mimic	4	14	18
Total		73	29	102

**Table 4:** Sensitivity, specificity, positive predictive value, and negative predictive value

	**SE (95% CI)**	**Sp (95% CI)**	**PPW (95% CI)**	**NPW (95% CI)**
Stroke vs. mimic	94% (86 to 98)	48% (29 to 67)	82% (72 to 89)	77% (52 to 93)

SE, sensitivity; Sp, specificity; PPW, positive predictive value; NPW, negative predictive value.

**Table 5:** Final diagnosis and etiology at discharge

**Final diagnosis (*n *** **= 102)**		**Territory**		**Etiology (TOAST)**	**Percentage**
TIA	*n* = 20	Anterior circulation	*n* = 58	Large artery atherosclerosis	50
Ischemic stroke	*n* = 50	Posterior circulation	*n* = 12	Cardioembolism	24
Hemorrhagic stroke	*n* = 3			Small vessel occlusion	13
Subdural hematoma	*n* = 2			Stroke of other etiology	4
No stroke	*n* = 27			Stroke of undetermined etiology	9

#### *Transcranial color-coded duplex sonography in the field*

Ultrasound contrast agents were administered in 41 patients (40%), and no adverse event was noted. Despite the use of UCA, inferior temporal bone windows were found in 11 of the 102 patients (11%) (in 5 patients bilaterally, in 6 patients unilaterally), and these were excluded from further analysis testing sensitivity and specificity of prehospital TCCS. An additional patient was excluded who presented with MCA occlusion with related hemiparesis and spontaneous thrombolysis during transport. One patient with a non-stroke diagnosis (temporal arteritis) and three patients with unremarkable neuroimaging findings yet stroke diagnosis at discharge were also excluded.

**Figure 4:**
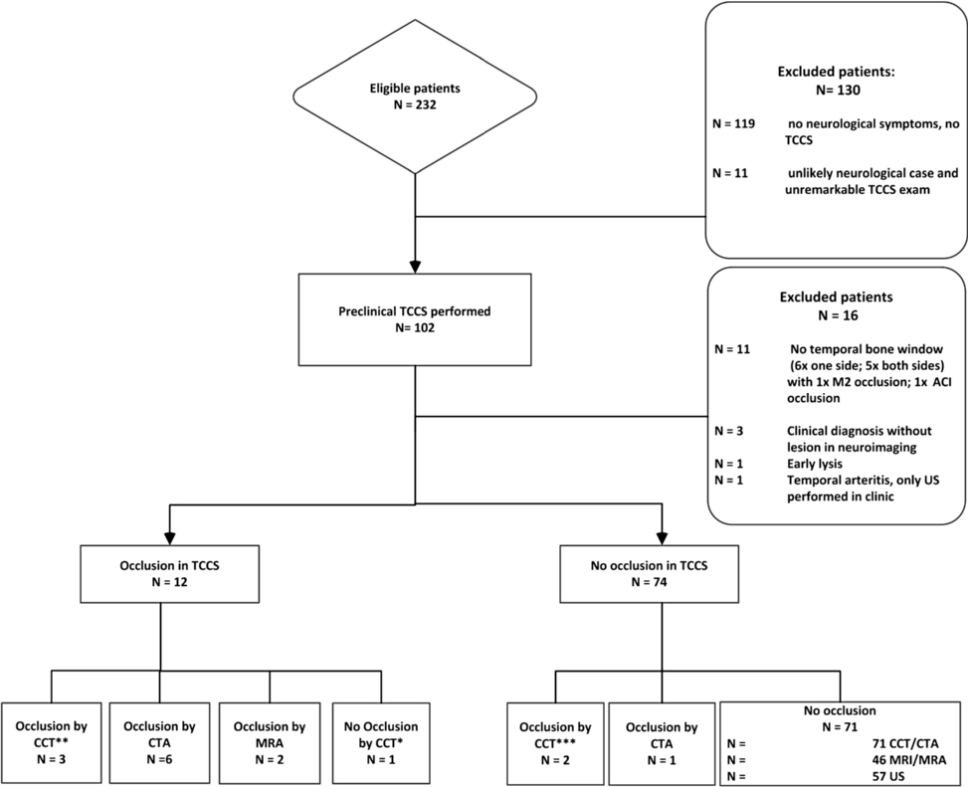
**Flow diagram showing the diagnostic imaging pathway used to diagnose ischemic stroke.** It includes the diagnostic accuracy for M1 and ICA pathology. US, ultrasound of intra- and extracranial arteries; TCCS, transcranial color-coded sonography; M1, middle cerebral artery mainstem; ICA, internal carotid artery.

The flow diagram (Figure 4) shows the diagnostic pathway and the neurovascular imaging reference methods obtained as ‘gold standard in hospital’. In 4% of patients, diagnosis of stroke was first detected by non-contrast CT (cerebral computed tomography (CCT)). In 7% of patients, CTA imaging first led to the final diagnosis.

**Figure 5:**
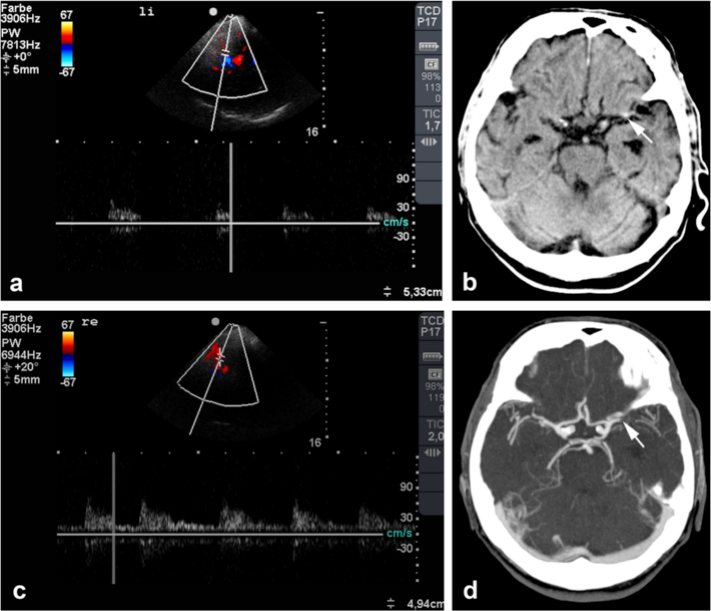
**Distal MCA mainstem occlusion in a patient with global aphasia and right-sided hemiparesis. (a)** Prehospital TCCS showed a resistance profile only at the origin of the left MCA, **(b)** whereas flow in the contralateral MCA was unremarkable. At the hospital, **(c)** a CT scan displayed a dense artery sign (arrow), and **(d)** a CTA showed occlusion of the MCA (arrow).

**Figure 6:**
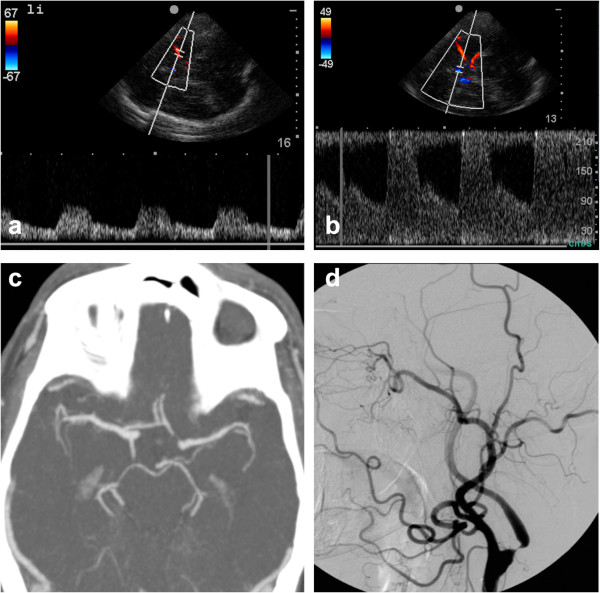
**Cross-filling suggestive of high-grade stenosis or occlusion of the ICA.** Images obtained in a 73-year-old patient suffering from a TIA with a 5-min-long paresis of the right leg. During transport, the patient's high blood pressure and angina pectoris prompted a decision to admit him to the Department of Cardiology. The results of the TCCS changed that decision, however, and the patient was admitted to the Stroke Unit. Surgery was performed the following day. **(a)** TCCS reveals normal flow in the left MCA. **(b)** Flow in the left ACA was increased and retrograde, suggesting collateral filling through the anterior communicating artery. **(c)** CT-angiography shows patency of all intracranial arteries but lacks flow information. **(d)** DSA on the same day confirms a tight, high-grade stenosis at the origin of the left ICA.

Preclinical TCCS demonstrated 12 occlusions or high-grade stenoses of major brain-supplying arteries (MCA and ICA) including 10 M1-MCA occlusions. Internal carotid artery (ICA) occlusions were diagnosed when reversed flow (‘cross-filling’) occurred in the ipsilateral ACA; this finding is indicative of >80% stenosis or total occlusion of the ICA according to the ECST criteria [24]. Standard imaging studies (CTA, MRA, and CCT) showed 14 major cerebral artery occlusions: 10 involving the MCA and 4 involving the ICA (Table 6). In the early days of the study, a PCA was mistaken to be a patent MCA in one patient when the UCA was incorrectly injected through a filter system, resulting in the destruction of microbubbles and inferior image quality. Also, TCCS resulted in the misdiagnosis of distal MCA occlusion in one patient, according to the Zanette index [22]. In this patient, an atypical parieto-occipital intracerebral hemorrhage (ICH) caused dislocation of the MCA, which led to a near-perpendicular angle of insonation. In retrospect, considering the lack of resistance in the low-flow profile and use of the UCA may have helped avoid the misdiagnosis (an example of a correct diagnosis of distal MCA occlusion is shown in Figure 5). Two >80% stenoses or total occlusions of the ICA were not detected; in those cases, the examiner investigated both MCA arteries according to the study protocol but did not examine the ACA and, therefore, missed a cross-filling phenomenon (Figure 6). In summary, we found a sensitivity of 90% and specificity of 98% (positive predictive value 90%, negative predictive value 98%) in achieving a correct diagnosis of MCA occlusion.

**Table 6:** Diagnostic accuracy TCCS

		**Standard stroke imaging**^**a**^		**Total**
		**Occlusion**	**No occlusion**	
MCA mainstem and ICA				
Preclinical TCCS	Occlusion	11	1^b^	12
	No occlusion	3^c^	71	74
Total		14	72	86
MCA occlusion				
Preclinical TCCS	Occlusion	9	1^b^	10
	No occlusion	1^d^	75	76
Total		10	76	86

^a^CTA, MRA, and CCT according to diagnostic pathway in stroke unite; ^b^massive intracerebral hemorrhage (ICH) described as distal MCA occlusion; ^c^no ACA imaged and therefore 2 ICA occlusions or >80% stenosis with cross-filling missed; ^d^contrast through filter, and PCA described instead of MCA occlusion. CTA, computed tomography angiography; MRA, magnetic resonance angiography; CCT, cerebral computed tomography; ACA, anterior cerebral artery; ICA, internal carotid artery; MCA, middle cerebral artery; PCA, posterior cerebral artery.

**Figure 7:**
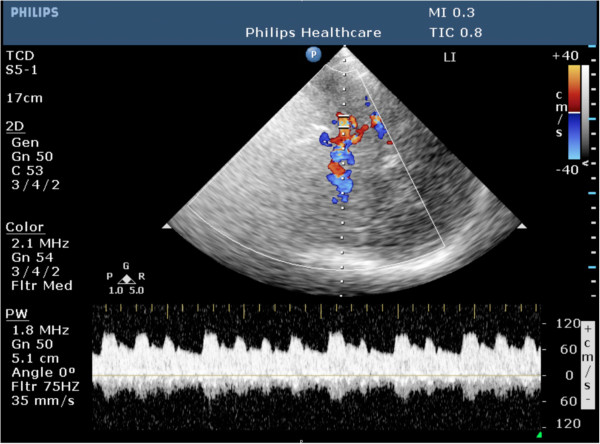
**TCCS with Doppler spectrum revealing tachyarrhythmia with changing cardiac output volumes in a 50-year-old patient.** The patient experienced 5 h of mild facial paralysis and weakness of the left arm. MRI confirmed a small cardioembolic right MCA infarction (not shown).

Patent MCAs, as demonstrated on standard neurovascular imaging studies (CTA, MRA) in the hospital, were diagnosed correctly in 75 of 76 cases in which an ischemia in any of the MCA territories was suspected and in 71 of 72 cases in which an ischemic stroke of the MCA-M_1_ and ICA was suspected (Table 6). One false-negative ICA stenosis could be identified on CTA scans. Two large hypodense areas indicated another false-negative ICA and an MCA mainstem occlusion. In one patient, in whom an atypical frontal ICH caused severe left-sided hemiparesis, a clinical syndrome potentially attributed to MCA occlusion, normal flow in the MCA was observed and confirmed by CTA. However, in addition to a diagnosis of major vessel occlusion, TCCS supported the likelihood of cerebral ischemia, as demonstrated in a patient in whom the first diagnosis was tachycardiac atrial fibrillation resulting in a changing peak systolic flow pattern (Figure 7).

### Discussion

In this study, we evaluated the potential of prehospital stroke assessment when an emergency doctor is joined at the scene of the event by a stroke neurologist equipped with a mobile ultrasound system for transcranial vascular diagnostics. More than half of the patients seen after a stroke emergency call were identified as not suffering a stroke by virtue of ‘neurological eyeballing’. The remaining patients who underwent combined neurological and neurosonological examinations were identified as having a stroke with a sensitivity of 94% and a specificity of 48%. This seemingly disappointing result is counterbalanced by high diagnostic sensitivity and specificity in patients with MCA occlusions (90% sensitivity and 98% specificity) or combined pathology in the anterior circulation for which the study was not designed (78% sensitivity and 98% specificity).

### Prehospital stroke diagnosis

The high percentage of patients (56%, 130 of 232 patients) who were excluded from the study because they had no neurological symptoms shows the difficulty faced by dispatch center staff in distinguishing between stroke and non-stroke symptoms based on a telephone call. In the current setting, an experienced neurologist performed an individual and focused neurological examination in addition to a neurovascular diagnosis in the emergency setting using TCCS. Further improvement may be added by systematically adding data of other findings (atrial fibrillation, list of medications, medical history) to combine to a probability model for stroke occurrence.

A delay of prehospital time is counterproductive in efficient stroke treatment. Therefore, the emergency teams were advised not to wait for the stroke mobile. Furthermore, examination and all associated acts were done during transport and at the site of initial treatment only if primary care was ongoing and not disturbed by neurological or ultrasound examination.

### Diagnostic accuracy of neurovascular imaging

Initiation of stroke-specific therapies first requires a battery of diagnostic tests with the major focus on excluding the presence of ICH. Here, sensitivity of CCT is beyond controversy [25]. Comparing the state-of-the-art stroke imaging modalities, MRI and diffusion weighted imaging (DWI) show the best sensitivity and specificity and DWI additionally has a very good predictive value for the ‘penumbra’ [26,27]. Unfortunately, in the real world, only 14% receive an MRI in the emergency department (ED) and only 29% within the first 12 h [28]. As CTA is not being obtained routinely, many physicians in EDs rely on CCT only. However, CCT alone shows a low sensitivity for ischemic stroke detection (sensitivity 27% to 64%) [29-32] and has a mean sensitivity for early infarction sign detection of 66% (20% to 87%) with a specificity of 87% (56% to 100%). One can argue however that ischemic stroke can be treated within the first 4.5 h of stroke onset using recombinant tissue plasminogen activator (rtPA on the basis of minimal imaging information (i.e., the exclusion of brain hemorrhage with CT), and therefore, the high sensitivity of CT for hemorrhage makes standard CT an important technique for the assessment of patients with acute stroke.

On the other hand, rtPA alone shows a low rate of acute recanalization particularly in proximal vessel occlusion (distal ICA 4.4%; M1-MCA (32.3%); M2-MCA (30.8%)) which is significantly improved only with an endovascular or combined (‘bridging’) approach [33-35]. Even though recent trials question the long-term benefit of an interventional approach in general, they on the one hand do show benefit for the subgroups we focused on (M1, carotid T) [36]. On the other hand, they stress the need for very early diagnosis, since even a 1-h delay in the time to treatment negates the benefit of a higher recanalization rate with endovascular treatment [37].

Bedside transcranial ultrasound has proved its high agreement with CTA studies, good identification of vessel occlusions amenable for interventional treatment, and good predictive values for the outcome of the patient in several studies [16,17,38].

In our study, TCCS in the field and with portable small ultrasound machines was performed with high accuracy by experienced investigators and showed an almost similar diagnostic accuracy compared with those of previously published studies performed in a hospital setting, in which blinded TCCS was compared to the reference method [14]. Yet, the variety of positions in which the patient had to be examined in different settings required substantial experience and dexterity on the part of the investigator, so in the future, some certification may be advised for this specific application.

### Prehospital stroke projects

A variety of prehospital stroke projects are currently underway. Examples include the Stroke Angel project [39], STEMO [40], Mobile Stroke Unit (MSU) [41], Aster (http://www.aster-magdeburg.de), and Med-on-@ix [42] projects. All these projects differ in concept, personnel required, timelines, and costs.

The current MSU concept aims to bring guideline-adherent stroke treatment directly to the emergency site using a specialized ambulance equipped with a CT scanner, point-of-care laboratory and a telemedicine connection to the hospital (ClinicalTrials.gov identifier: NCT00792220). They could significantly reduce the time from alarm to therapy decision from 76 to 35 min in the MSU group. However, distances to hospital differ (6 km (4 to 10) vs. 8 km (6 to 15) in control group) and are much shorter than in our study (10 km (2 to 41). Unavailable diagnostic equipment in 18% (22 of 122) due to technical problems with the CT scanner or the laboratory was comparable to 10% exclusions due to insufficient temporal bone window. Bringing CT diagnostic to the patient is, from our point of view, not applicable in rural areas due to long distances and extensive costs.

A study related to MSU represents the Phantom-S Study [43]. Their aim is to reduce alarm-to-needle time by the implementation of a CT scanner, teleradiological support, and point-of-care-laboratory-equipped ambulance. In comparison to our study, the covered region is limited to a maximum of 16 min from dispatch to arrival at the patient within central Berlin. Furthermore, the ambulance staff was specially trained for the study, whereas we implemented our study within normal routine emergency medical services (EMS). This approach will be most effective within urban centers.

Compared to others, we focused on ischemic stroke of proximal M1/M2 occlusions leading to the clinically worst outcome but with relatively good prognosis if treated by experts timely and therefore crucial to be detected and streamlined to a specialized stroke unit early [33]. In comparison to other projects, our focus was not only on metropolitan areas but also on rural areas with long distances to the next stroke unit (Figure 1). We found that in-field use of mobile ultrasound systems does not result in prolonged prehospital delays and is particularly suited in patients with large arterial occlusions. Mobile ultrasound showed an overall sensitivity of 78% and specificity 98% for ‘major MCA or ICA’ occlusions. Our primary aim in this study was to detect vessel occlusions and not to exclude brain hemorrhage with the use of ultrasound. In order to initiate, i.e., thrombolysis cases of hemorrhagic stroke and stroke mimics (Tables 2, 3, and 4) must be excluded and must still require profound diagnostics at specialized stroke units. Despite a high likelihood of ischemic stroke in cases of MCA occlusion detected by prehospital TCCS, additional tools such as point-of-care serum stroke diagnostics are needed in the future before prehospital thrombolysis.

Another aspect of prehospital cerebrovascular diagnostics is the lack of comparative prehospital data. Neither data on diagnostic accuracy nor the percentage of CTAs performed in the publications of other mobile stroke units using a CT scanner is available; thus, comparison of diagnostic accuracy of prehospital TCCS is limited.

The combination of the different experiences gained by the various preclinical stroke treatment studies could be used to implement a reliable telemedical tool to detect and treat stroke patients in a countrywide manner.

### Limitations of the study

The limitations of our study are the relatively low number of proximal large vessel pathology (MCA and ICA) and no standardized stroke neurovascular diagnostic algorithm in the two receiving stroke units. The latter is due to the fact that stroke treatment has different diagnostic algorithms depending on a variety of factors such as time window, age, and availability of stroke MRI, among others - a drawback common in health-care research. Furthermore, we did not pursue contact with the patients we ‘eyeballed’ as suffering from something other than stroke and thus did not compare our initial prehospital diagnosis with the final diagnosis in these patients. We examined fewer than half of our patients using the contrast agent thus explaining the high number of patients with insufficient temporal bone windows (11%). The study concept did not require to always examine the complete circle of Willis in all patients, leading to two missed >80% ICA stenosis with cross-filling phenomena documented in stroke unit TCCS. A further limitation is that findings from ultrasound were compared to those from CTA or MRA on a later stage in time. In practice, this limitation is almost impossible to overcome, but during the time between both investigations, the situation potentially may have changed which could influence the calculated relationship between both techniques. We have since implemented several improvements in our ongoing study protocol.

### Outlook

Resources in health care are limited, and prehospital stroke projects will ultimately be assessed from a socioeconomic point of view [8,44]. If current technical problems can be solved, telemedicine might offer a rapid transfer of stroke expertise to EMS [45].

In Germany, TCCS has already been successfully performed by medical technicians under the supervision of a neurologist. Provided specific stroke training, paramedics may be able, in the future, to perform TCCS and transfer these data to a vascular neurologist for interpretation. Mikulik et al. [38] showed in a pilot study that an inexperienced health-care provider (for example paramedics) could perform a bedside examination guided by an experienced neurosonographer via telemedicine. The use of a combined neurological TCCS examination may also be extended to situations in which helicopter emergency transport is necessary, a form of transport already shown to provide the highest rates of thrombolysis in stroke patients in an Austrian study [46].

The TEMPiS project has shown to deliver high experienced stroke therapy to underserved areas and proved cost-effectiveness in hospital settings [47]. A consequent continuation of our project would be to transfer clinical and ultrasound data, obtained by regular emergency personal during the prehospital examination, to experienced stroke neurologists. The use of probability algorithms for a stroke diagnosis combined with the neurovascular status of the patient may lead to high-quality telemedical interactions between paramedics and emergency physicians on the one hand and specialists at stroke units on the other [39,42,47,48].

## Conclusions

Our study demonstrates the feasibility and high diagnostic accuracy of emergency neurological examinations that include the use of mobile transcranial ultrasound systems to assess the cerebral circulation. At this point, the accuracy of stroke diagnosis is dependent on the expertise of stroke neurologists, including their ability to perform TCCS in a variety of situations and to correlate the results to patients' neurological symptoms. However, with telemedical support, administration of UCA, and specific stroke training for paramedics, this system may be feasible for broad application, including rural areas where the choice of treatment may currently be more limited due to long prehospital delays.

## Competing interests

The authors declare that they have no competing interests.

## Authors' contributions

MH performed data analysis and drafted the manuscript; SB acquired clinical data, participated in the study design and drafted and corrected the manuscript; TH participated in the study design and corrected the manuscript; ME acquired clinical data; MZ participated in the study design; KPI participated in the study design; JP contributed clinical data and was involved in logistics; HP contributed clinical data as clinical collaborator; UB corrected the manuscript and was involved in the study design; FS acquired clinical data, participated in the study design and drafted and corrected the manuscript. All authors read and approved the final manuscript.
